# Outstanding Geoscientific Sites in Periurban Areas: the Case of Roses Lighthouse Geosite (Cap de Creus, eastern Pyrenees)

**DOI:** 10.1007/s12371-023-00847-4

**Published:** 2023-05-26

**Authors:** Elena Druguet, Jordi Carreras, Marina Cervera, Josep Mercadé, Jèssica Espasa

**Affiliations:** 1 Departament de Geologia, Universitat Autònoma de Barcelona, Bellaterra, 08193 Barcelona, Spain; 2 Sa Tórtora 8, Cadaqués, 17488 Girona, Spain; 3 Departament d’Urbanisme I Ordenació del Territori, Universitat Politècnica de Catalunya, Campus Sud Avgda. Diagonal 649, 08028 Barcelona, Spain; 4 Departament d’Enginyeria Civil I Ambiental, Universitat Politècnica de Catalunya, C. Jordi Girona, 1-3, 08034 Barcelona, Spain; 5 iMuntanya, Suspended Footbridges Experts, Polígon Industrial Els Plans, Carretera Castellnou N-II, Castellnou de Seana, 25265 Lleida, Spain

**Keywords:** Geoconservation, Geotourism, Granitoid, Landscape architecture, Restoration, Shear zone

## Abstract

The coastal cliffs around the Roses Lighthouse (Cap de Creus, Mediterranean Costa Brava) display deformation structures generated during the emplacement of a syntectonic granodiorite and associated rocks (quartzdiorite enclaves and leucocratic dykes). These rocks were subjected to shearing and spectacular shear zones are present, which have been object of several scientific publications. The outcrops are considered of international high scientific value, being regularly visited by researchers and students from several European universities. In 2005, the site was included in the *Geosite Inventory of Catalonia*, but it does not have any special protection yet, despite decades of efforts to claim the need for protection and conservation in front of the constant deterioration and loss of outcrops due to strong urban and touristic pressure. A project of restoration, access improvement, and dissemination of geological values was finally executed between 2020 and 2021. The Roses case study leads us to the remark that urban and periurban geosites offer a good opportunity for promoting geological research, education, and tourism, provided its protection based on geoconservation criteria and a strong sustainable conservation management plan.

## Introduction

Geological heritage is many times located in areas of non-remarkable aesthetical interest or landscape value such as road cuts, quarries, urbanized zones, and other types of anthropized spots. Urban and periurban geoscientific sites fall into this category of geoheritage which is often severely threatened and whose conservation management plans can result very complicated to implement. This is due to the sum of two main factors:

1. The high anthropogenic pressure of these spaces in terms of urbanization and human impact (Rodrigues et al. 2011; Kong et al. 2020), especially if they represent sites of passage or touristic spots,
2. The still prevailing conservation policies which misleadingly assume that geological elements can only be associated to natural sites, thus excluding non-pristine localities from protection (Theodossiou-Drandaki 1998; Carreras and Druguet 2000).

However, in a similar way, as it happens with cultural-historical heritage, elements of geoscientific interest are independent of the characteristics of the exposure site, and contrarily to some currently established rules, they deserve protection whatever their environmental conditions are (Carreras and Druguet 2000; Brilha 2016). Furthermore, geoscientific heritage may be equally relevant to human culture as, for instance, building and ornamental stones heritage (De Wever et al. 2017; Pijet-Migon and Migon 2022).

Urban geology and geoheritage have long been documented (e.g., Bennett et al. 1996). In the last decade, we have seen a substantial increasing interest in promoting and disseminating geological values in urban and periurban areas, as reflected in a large number of publications by the geoconservation research community (e.g., Rodrigues et al. 2011; Del Monte et al. 2013; Reynard et al. 2017; Palacio-Prieto 2015; Petrović 2017; Habibi et al. 2018; Leguizamón et al. 2018; Capdevila-Werning 2020; Vegas and Díez-Herrero 2021). Most of these works agree in the diagnosis that protection of these spaces requires changing some prevailing strategies, eradicating the restrictive criteria of wilderness, and further exploring the relationships between geoheritage and cultural heritage (Pijet-Migon and Migon 2022; Wolniewicz 2022). Once these areas have been recognized and protected as assets of scientific interest, the fact that they are in publicly accessible zones can be considered as a good opportunity to make visible and disseminate geological heritage to society. This can be done through environmental education and geotourism, provided that such tourist use is sustainable and in balance with geoconservation needs (Newsome and Dowling 2018; Kubalíková et al. 2021).

In this paper, we present a case study of an outstanding geoscientific site at Roses (Cap de Creus peninsula, Eastern Pyrenees) which has been recently restored after decades of gradual degradation due to urban pressure.

## The Geological Heritage of Roses Lighthouse

**Fig. 1:**
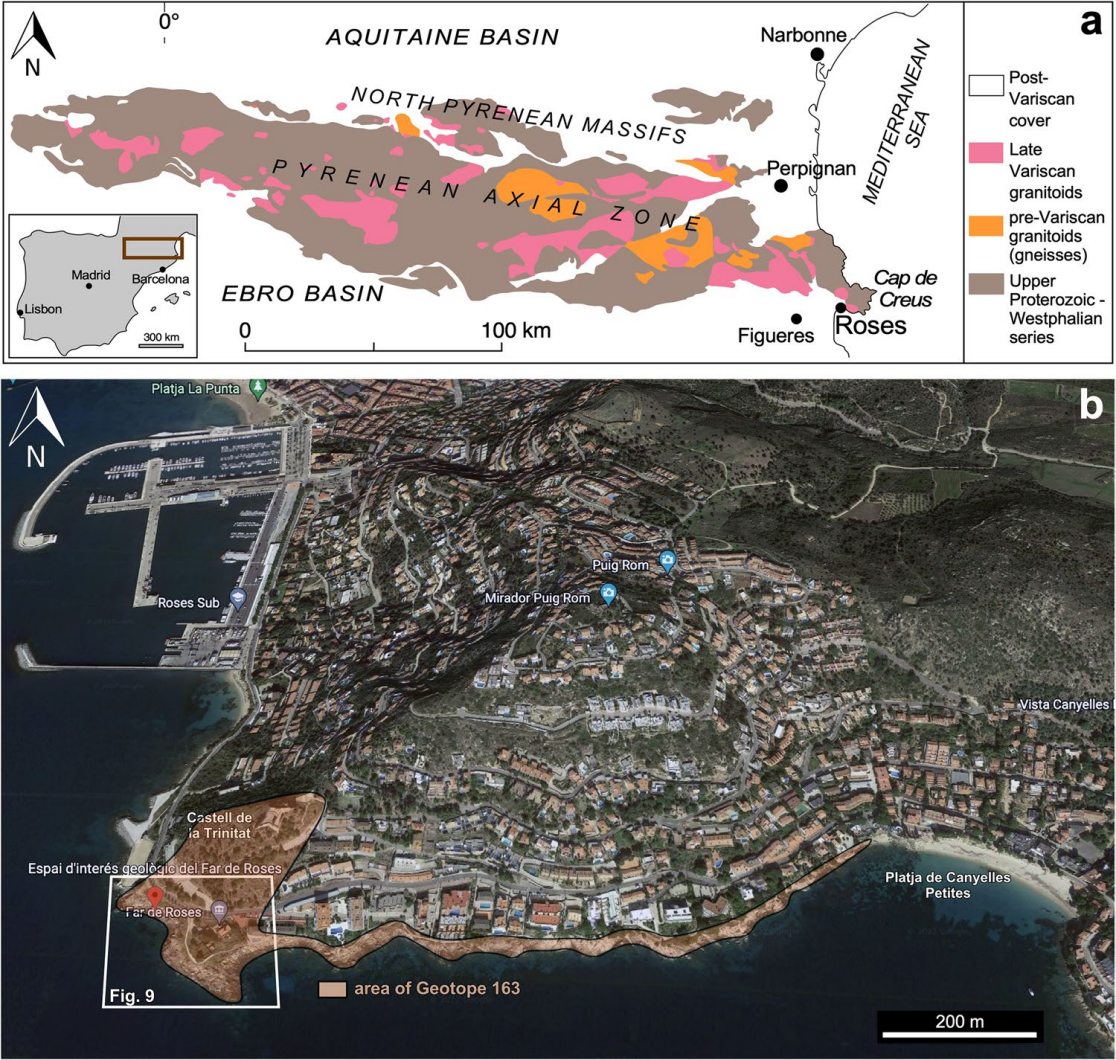
**a** Geological setting of the Roses area at the scale of the Pyrenean chain (modified after Druguet et al. 2014). **b** Geographical setting of the Roses Lighthouse geosite (Google Earth 3D view)

The outcrops are located in the southwestern coast of the Cap de Creus massif (northern Costa Brava and easternmost end of the Pyrenean Range) along a narrow rocky coastal fringe in the periurban area of Roses (Fig. 1). The site, whose only instrument of protection is that of being integrated into the maritime-terrestrial public zone (abbreviated, ZMT), has for long been submitted to strong touristic pressure.

**Fig. 2:**
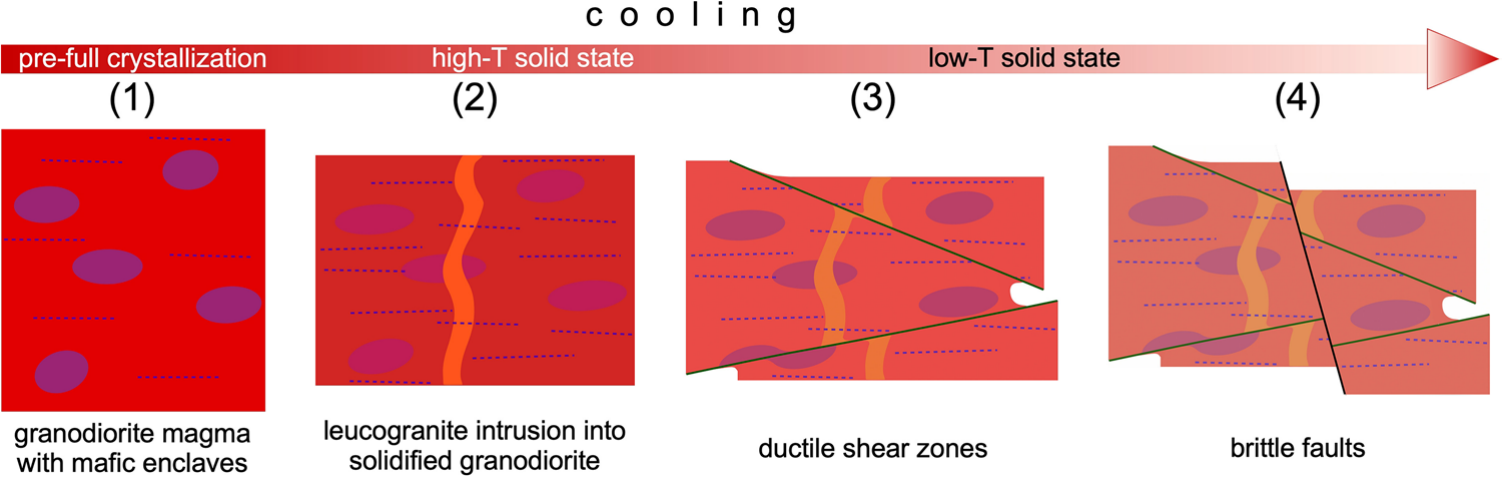
Sketches of the evolution of the Roses pluton (see text for explanations)

**Fig. 3:**
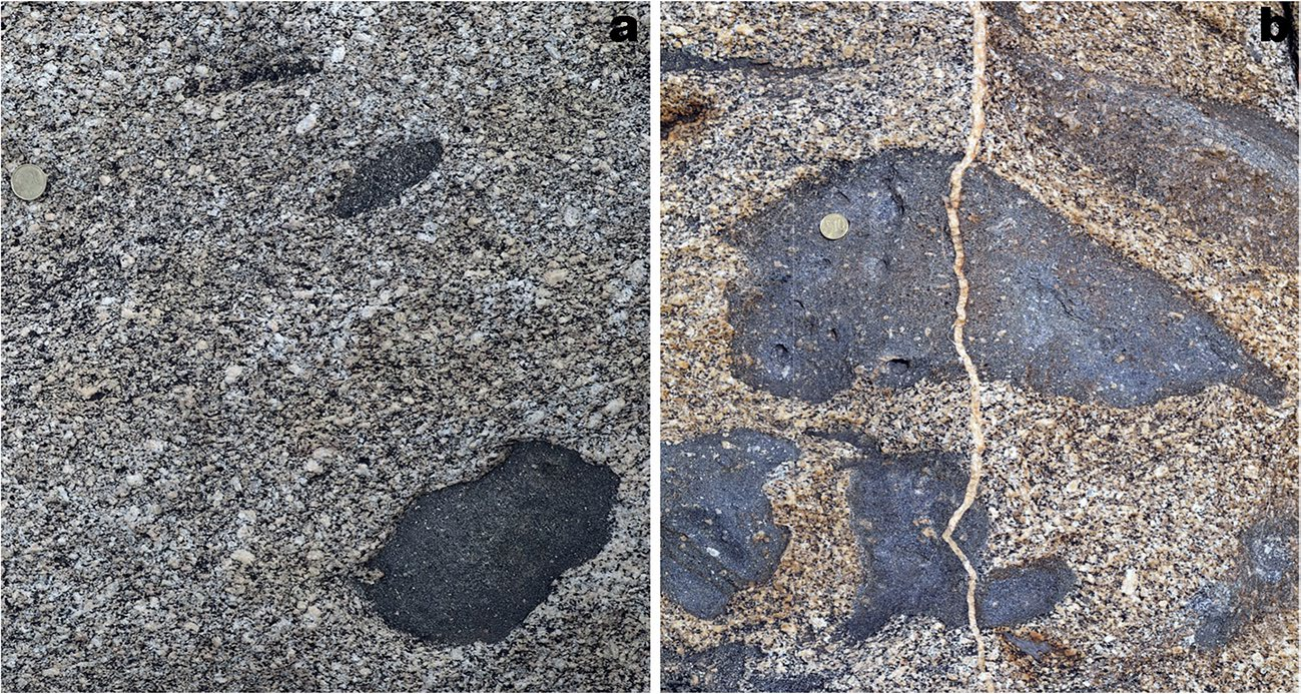
**a** Quartzdiorite enclaves in mid- to coarse-grained granodiorite. The magmatic fabric is defined by the preferred orientation of grains in the granodiorite and elongation of the enclaves. **b** Aplite vein cross-cutting the granodiorite and the mafic enclaves

**Fig. 4:**
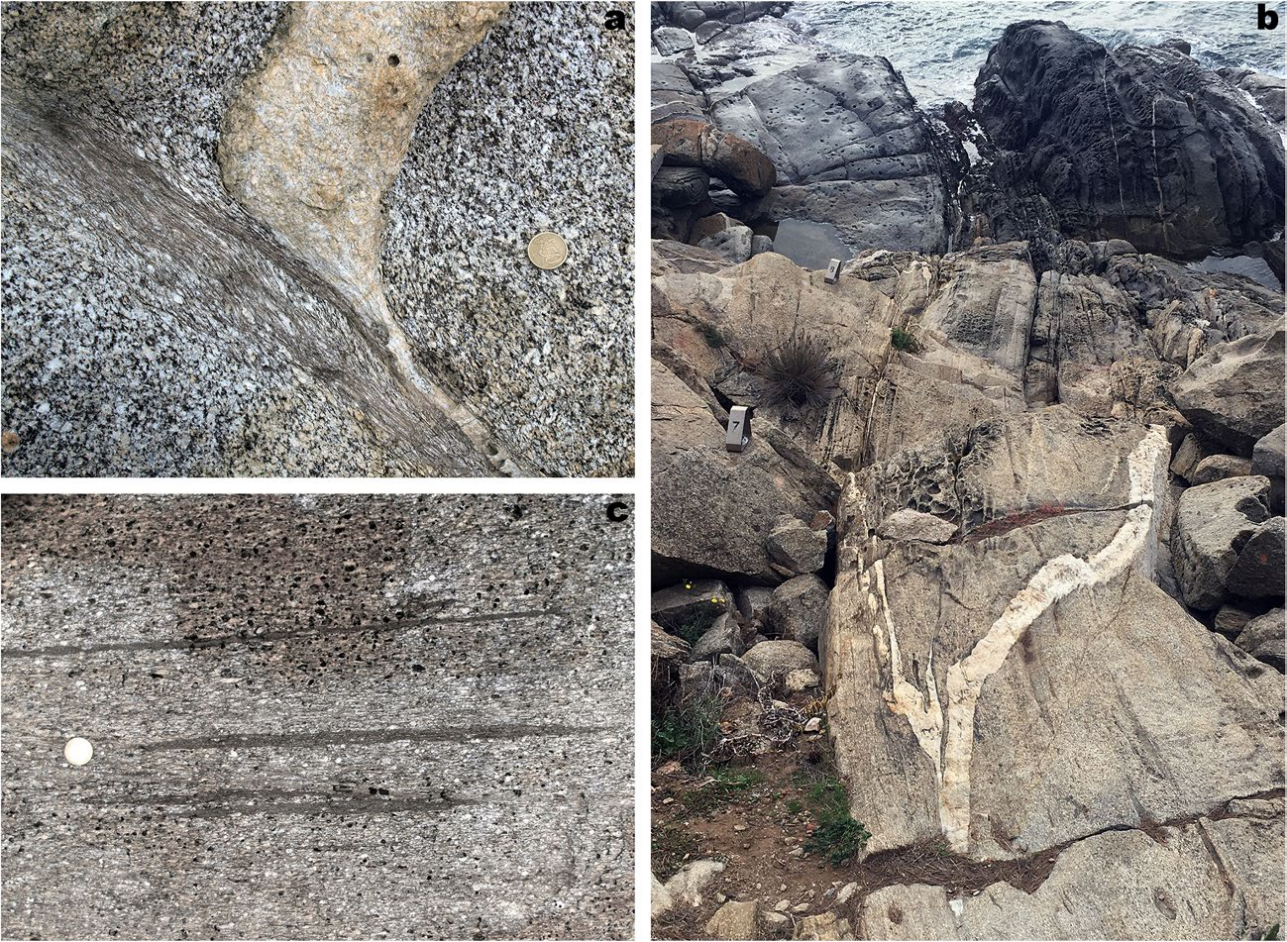
**a** Ductile shear zone affecting the Roses granodiorite and a leucocratic dyke (point of geological interest #3 of the geological itinerary). **b** View of points of geological interest #7 and #8 of the geological itinerary, located right below the lighthouse. The effects of a large shear zone on the granodiorite can be observed, especially on a deformed light-colored vein, which is folded and stretched. **c** Detail of extremely elongated mafic enclaves within the mylonitic band shown in **b**. See location of points of geological interest in Fig. 9

**Fig. 5:**
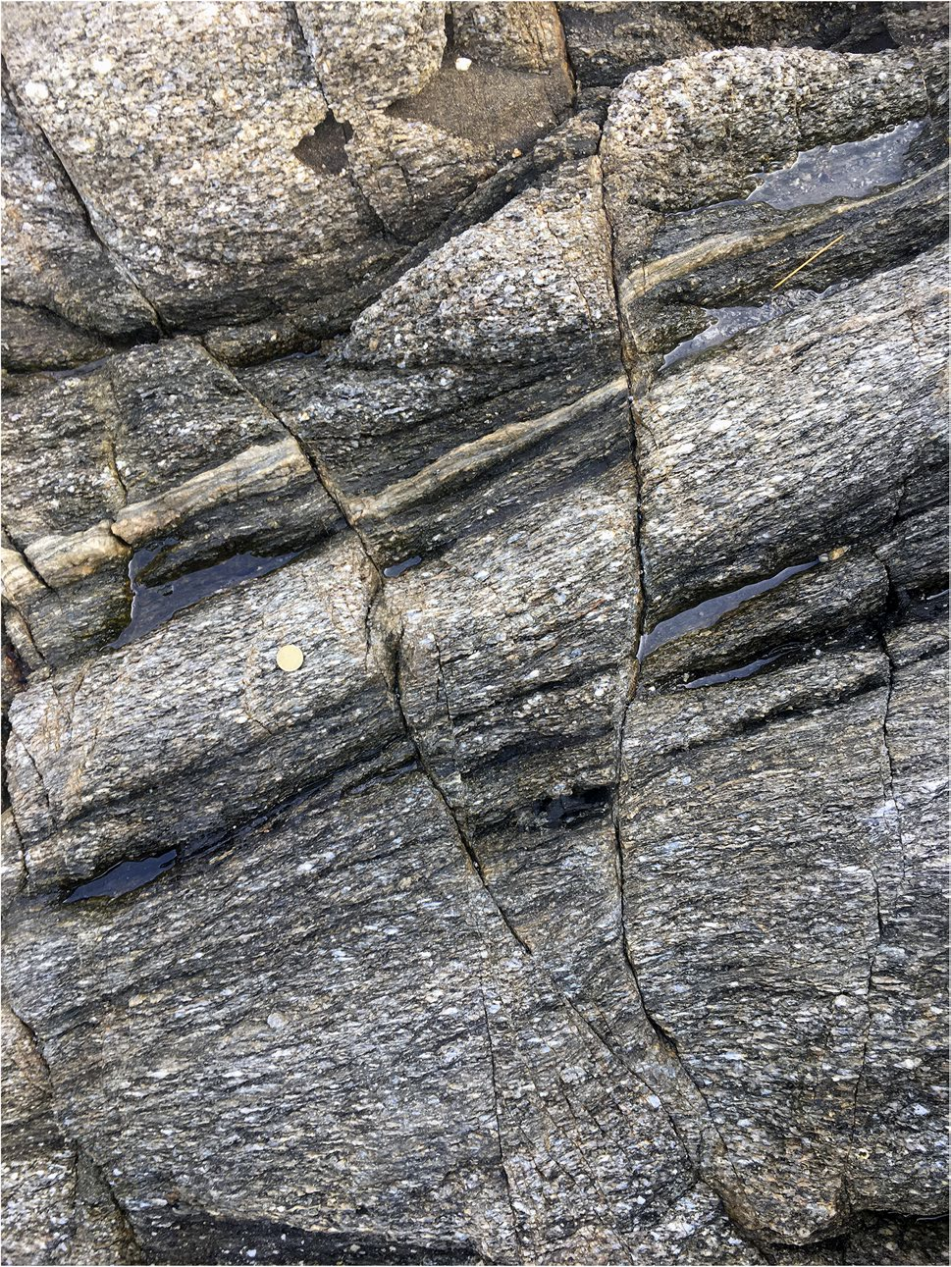
Conjugate brittle faults cross-cutting the mylonitic granodiorite

The rock exposures on the coastal cliffs around the Roses Lighthouse display deformation structures generated during the syntectonic emplacement and progressive cooling of the Roses pluton (Fig. 1a) at the end of the Variscan orogeny. Plutonic rocks are represented by granodiorite (dated at 291 ± 3 Ma, Druguet et al. 2014) and associated rocks (quartzdiorite enclaves, and leucocratic dykes). Detailed analysis of the structures allows to establish a continuous deformation history for the Roses pluton, which can be summarized in four main stages (Fig. 2; Carreras et al. 2004; Carreras and Druguet 2013):

1. At the first stages of emplacement, when the granodiorite was still partially molten, a rather homogeneous magmatic foliation developed because of coeval deformation and magmatic flow. Such foliation is defined by the preferred orientation of feldspar and biotite crystals. At this stage, quartzdiorite enclaves became elongated sub-parallel to the foliation (Fig. 3a).
2. Ongoing deformation at high-temperature solid state allowed the emplacement and solidification of residual magmas along fractures in the form of dykes and veins of aplite and pegmatite (Fig. 3b).
3. At lower temperature conditions (when the pluton was completely solidified), deformation progressively localized, giving rise to a network of predominantly sinistral and minor dextral ductile shear zones. Spectacular exposures of cm- to decametric-sized shear zones and associated mylonitic rocks are present (Carreras and Losantos 1982; Simpson et al. 1982; Simpson 1983; Carreras et al. 2004; Montomoli et al. 2008; Carreras and Druguet 2013; Fig. 4).
4. At the end of the same progressive deformation event, strike-slip faults and a network of joints developed at clearly brittle conditions (Fig. 5).

**Fig. 6:**
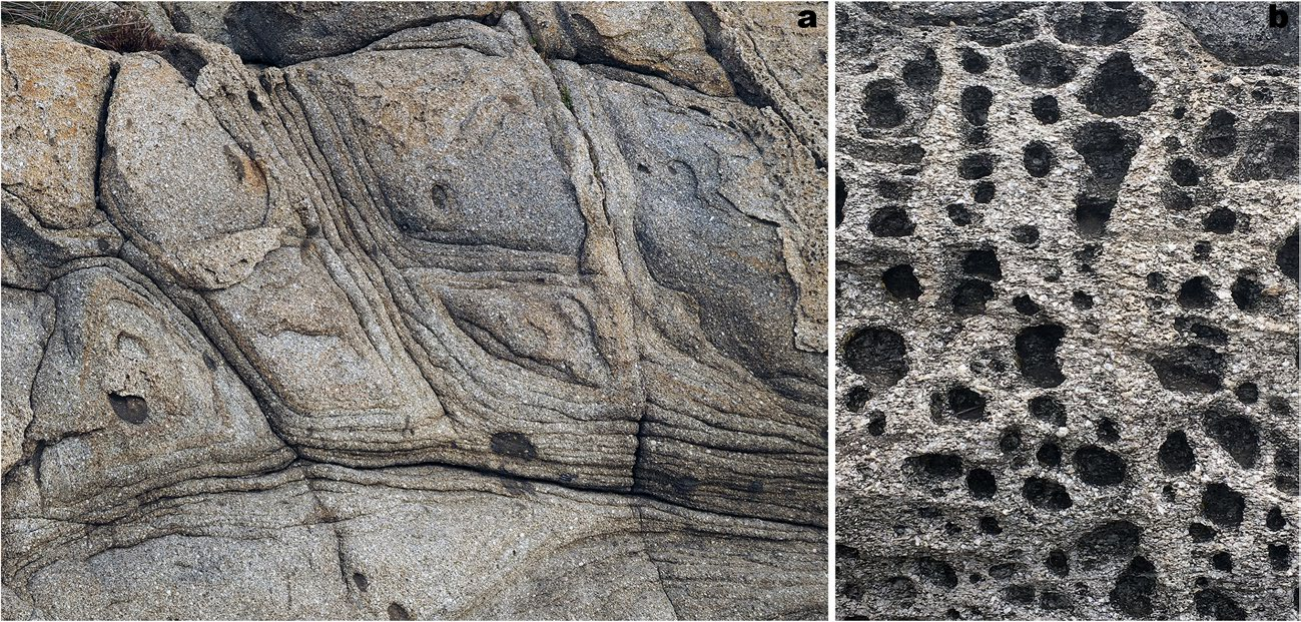
Examples of weathering forms: onion skin-type spheroidal disjunctions with evidence of joint-control (**a**) and honeycomb-type tafoni (**b**)

After uplift and denudation of the Cap de Creus massif, the geological evolution concludes in the Quaternary with weathering and erosion of the crystalline rocks. Planar exfoliation, spheroidal desquamation, and tafone are the most prominent landforms that shape this coastal geosite (Fig. 6).

The described structures highlight the excellence of these outcrops in the fields of tectonics and petrology, particularly the exceptional character of the ductile and brittle-ductile shear zones, according to their representativeness, uniqueness, and scientific value.

This area, together with those on the northern coast of the Cap de Creus peninsula (under legal protection since 1998), includes the first shear zones and associated mylonitic rocks that were recognized in Catalonia in the early 1970s. In 1979, an International Conference on Shear Zones in Rocks took place in Barcelona and Cap de Creus, attended by specialists from all over the world. In the early 1980s, visitors (geology students and researchers) and publications (Carreras and Losantos 1982; Simpson et al. 1982) made these outcrops famous among the structural geology international community. There are several publications that take examples from this locality (e.g., the seminal textbook of Ramsay and Lisle 2000; Platt and Passchier 2016). The visit to this locality is included in field trips linked to geological congresses (e.g., 2011 Geological Society of America Penrose Conference “Deformation Localization in Rocks”), workshops (e.g., 2006 First TecTask-IUGS Field Workshop on Structural Analysis), and in excursions of graduate and undergraduate Earth Sciences students from various Spanish and European universities. The Roses geosite is currently worldwide considered as a top classic locality in the fields of structural geology and tectonics.

These high scientific and educational values of Roses geosite are complemented by the on-site presence of remarkable geomorphological features (Fig. 6) and by proximity to the geo-, bio-, and cultural heritage of the Cap de Creus Natural Park. Furthermore, the locality is adjacent to some historical-cultural elements such as the Roses Lighthouse and the Trinity Castle (Castell de la Trinitat, Fig. 1). Given its closeness to Roses city, the suitability of the Roses geosite as a research, educational, and touristic resource is further enhanced by its good accessibility conditions and cultural-touristic attractions. Due to its Mediterranean climate, it also benefits from good seasonal/weather conditions prevailing during most of the year.

## Threats and Impasses on Protection of the Geosite

By the time the structures displayed in Roses were first described (early 1970s), outcrops were clean and did not present invasive vegetation or debris.

Despite the worldwide recognition acquired by the geosite and the evidences of progressive deterioration due to increasing urban pressure, legislation and conservation policies based on the protection of “natural elements” lead to the exclusion of the Roses Lighthouse site from the Cap de Creus Natural Park designated in 1998 as a protected area (Carreras and Druguet 1997; 2000).

Thus, the only real instrument of protection is that of being integrated into the maritime-terrestrial public zone (the narrow strip of land that can be affected by the sea waves during strong storms, abbreviated ZMT), which is subjected to restrictions on private use and building.

Researchers who had visited the area became concerned of the dangers and agreed that recovering these outcrops was an obligation. Here are some comments from geoscientists regarding the geological relevance of the Roses outcrops and the need for their protection:

- *The elegant outcrops at Roses show more features idealised in “textbook” shear zones than any others I have seen in Scotland, the Alps or elsewhere* (Dr. John Wheeler, Liverpool University).
- *I have no doubt that the loss of this area would be a great disaster for science. This area should be preserved and protected for international science* (Dr. Donald Hutton, University of Birmingham).
- *The magnificent shear zones observable on the coast of Roses are at risk of becoming inaccessible to observation in the very short term; some have already disappeared due to excessive urbanization* (Prof. Gerard Bossière, Université de Nantes).
- *The exceptional exposures display the best exposed and most accessible examples of ductile shear zones that I have encountered in my nearly 30 years of field research and excursions. For geologists, nothing can replace actually visiting classic localities and observing the rocks and structures first-hand* (Prof. Darrel S. Cowan, University of Washington, Seattle).

**Fig. 7:**
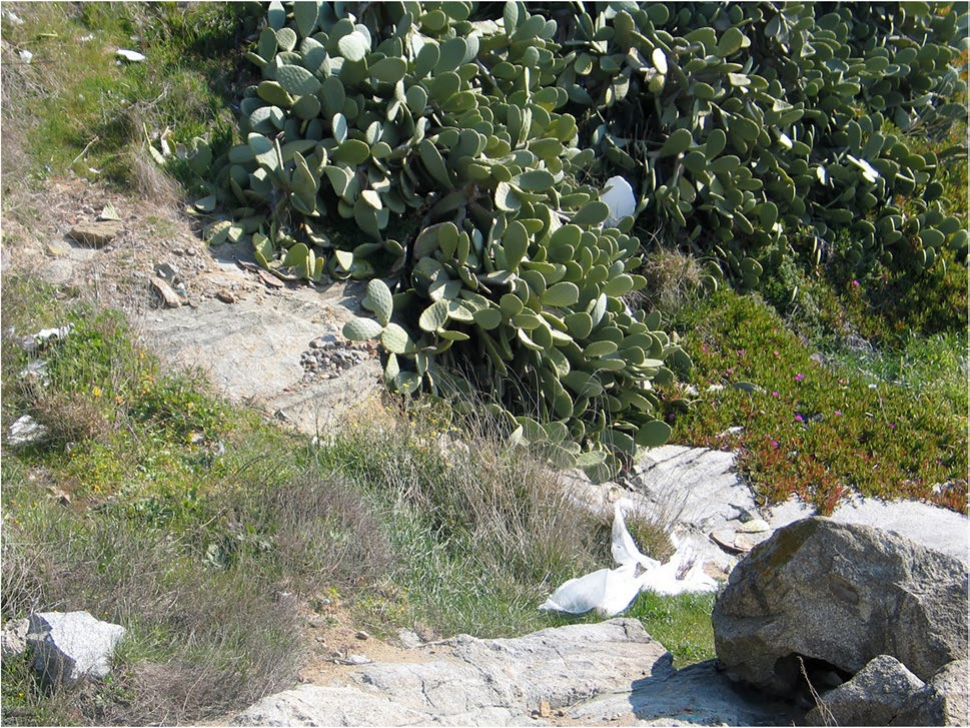
One of the nicer outcrops of ductile shear zones and mylonitic rocks became almost completely covered by invasive vegetation, debris, and garbage (photograph of year 2005)

However, urbanization of the area proceeded, and a coastal pathway was built in the early 2000s, causing partial hiding of some relevant exposures due to the accumulation of gravel and the growth of invasive plants (e.g., *Carpobrotus edulis*, see Fig. 7). In addition, the access was highly difficult due to the presence of agaves and pear cactus.

In 2005, the Roses Lighthouse site was included in the *Geosite Inventory of Catalonia* (Geotope 163 of Carreras and Druguet 2005, Fig. 1b), being catalogued as a very relevant site in the fields of structural geology and igneous petrology and for the interpretation of endogenous geodynamic processes. A high degree of degradation and vulnerability of the site was also reported in the inventory. However, this action did not involve any figure of legal protection.

## Restoration and Dissemination Project

**Fig. 8:**
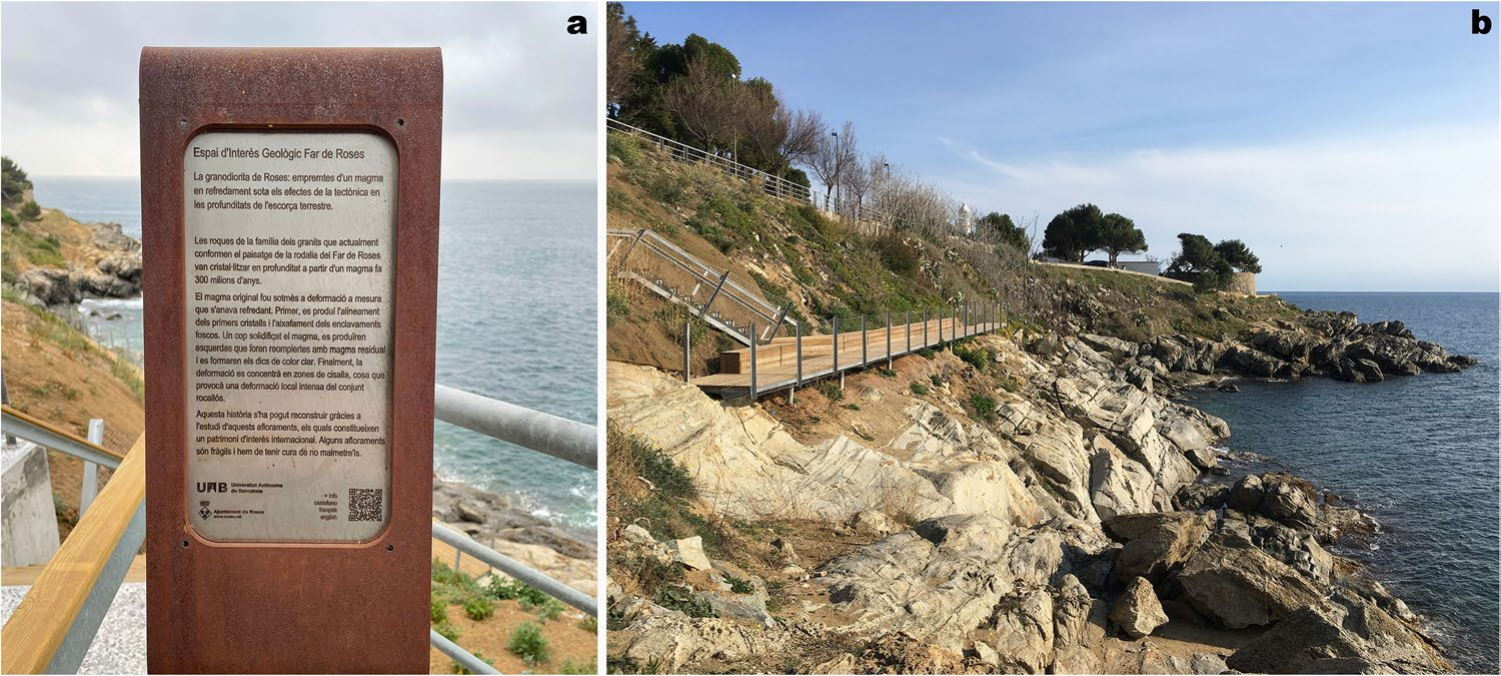
**a** Panel at the starting point of the “Site of Geological Interest Roses Lighthouse”. It includes a brief description in Catalan of the geological values (translated into English below) and a QR code to access to further information available at the Roses Municipality webpage: http://rosespedia.cat/index.php?title=Espai_d%27interès_Geològic_Far_de_Roses. *The Roses Granodiorite: Fingerprints of magma cooling under the effects of tectonics in the depths of the Earth’s crust. The granite-type rocks, which are now forming the landscape around the Roses Lighthouse, crystallized in depth from magma 300 million years ago. The original magma became deformed during cooling. The alignment of the first crystals and the flattening of the dark enclaves took place in the initial stage. Once the magma got solidified, cracks opened in the rocks and were filled with residual magma, forming the light-colored dykes. Finally, deformation concentrated in shear zones, causing an intense local deformation of the rock set. This story has been reconstructed thanks to the study of these visible outcrops, which comprise a heritage of international value. Some outcrops are vulnerable, so we must take care not to damage them.* **b** Panoramic view of part of the geosite with the newly built walkway (marked in Fig. 9)

**Fig. 9:**
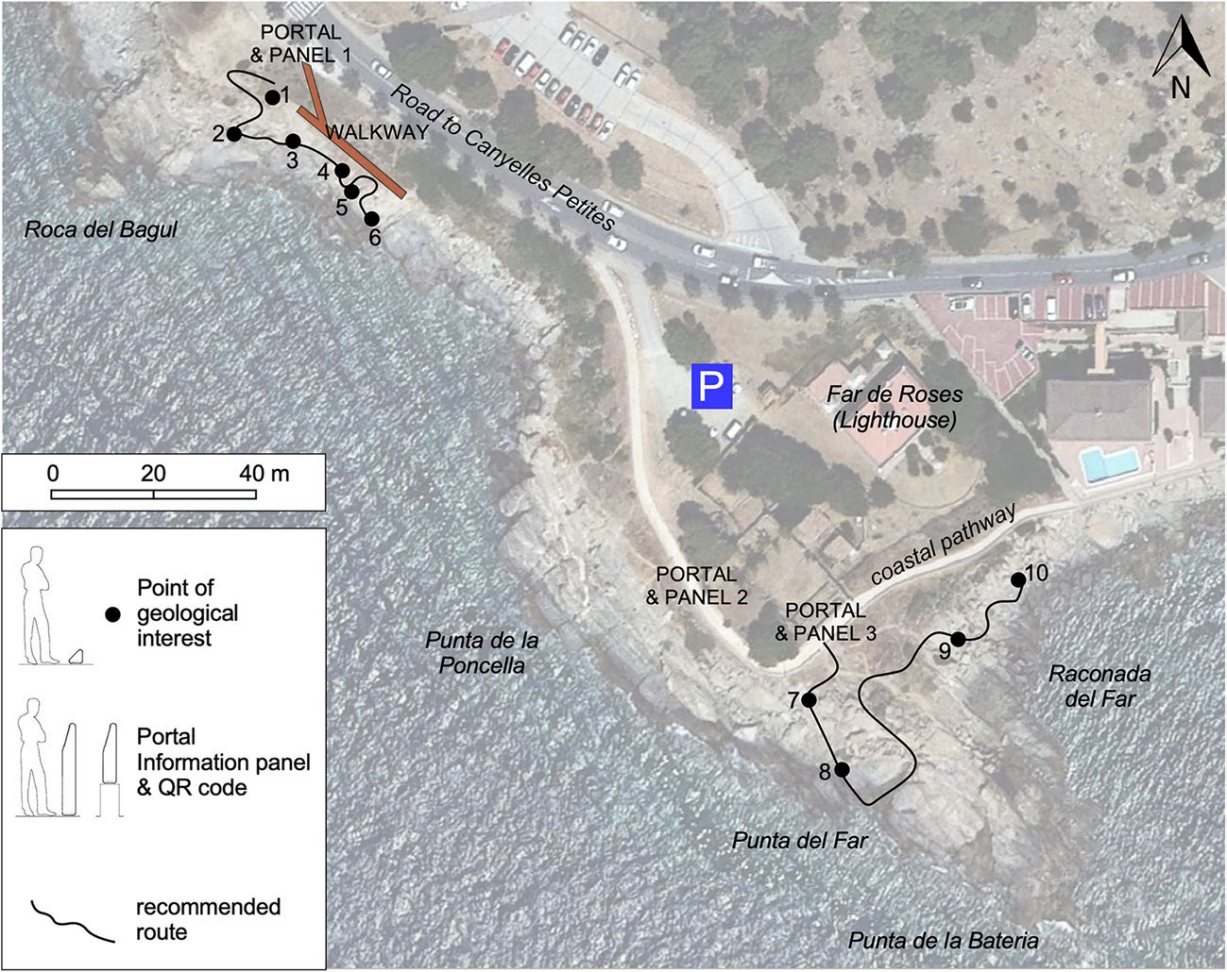
Map of the Roses Lighthouse geosite with indication of the main elements of the restoration and dissemination project. P: parking lot with space for ca. 10 vehicles. Base orthophotography from OrtoXpres 1.0 ICGC—Institut Cartogràfic i Geològic de Catalunya (2021). See location in Fig. 1

Thereafter, several projects for outcrops recovering were presented to the municipality of Roses. Finally, a restoration project was approved in 2019, and the works finished in May 2021, after 1 year of delay due to the COVID-19 pandemic. The area where the action was taken is smaller than that of the Geotope 163 of the 2005 *Geosite Inventory of Catalonia* (Fig. 1b), due to financial limitations and to the fact that part of the Geotope 163 is already within the Trinity Castle heritage domain. However, it includes the outcrops of greater scientific importance. The restored area has been named “Site of Geological Interest Roses Lighthouse” or “Roses Lighthouse Geosite”. The project, which was led by the architects, engineers and geologists coauthors of this article, aimed three main objectives:

1. Recover outcrop exposures by removal of plants, blocks, and gravel which had gradually hidden some of the outstanding structures.
2. Facilitate accessibility from the road and coastal pathway and a viewpoint, for which a walkway consisting of a staircase and a platform were designed and constructed (Figs. 8 and 9; see http://www.imuntanya.com/en/blog-en/the-roses-walkway-brings-us-closer-to-geological-heritage/). They are made of a wood-covered steel structure built on the slope next to the outcrops whose access was formerly very difficult.
3. Make divulgation actions to bring the geological values to the general non-specialized public. Three information panels and QR codes along a newly designed geological itinerary are included (Figs. 8 and 9). The itinerary includes 10 different points of geostructural interest accessible through three entrances (here referred as portals). Points 1–6 can be accessed from portal 1 and points 7–10 from portals 2 or 3. All the information is available online through the *Rosespèdia* webpage (http://rosespedia.cat/index.php?title=Espai_d%27interès_Geològic_Far_de_Roses), which is part of the Roses cultural heritage signaling project carried out by the Roses City Council. A more specialized, larger itinerary (from Roses Lighthouse to Platja de Canyelles Petites, Fig. 1b) had already been published by Carreras and Druguet (2013).

## Reassessing Geoheritage and Geoconservation of Roses Lighthouse

In this section, a comparative assessment of the geosite before and after restoration and dissemination works (performed in the period 2020- 2021) is attempted with the purpose of highlighting whether the pursued goals have been achieved and which issues should be improved through further management actions.

**Fig. 10:**
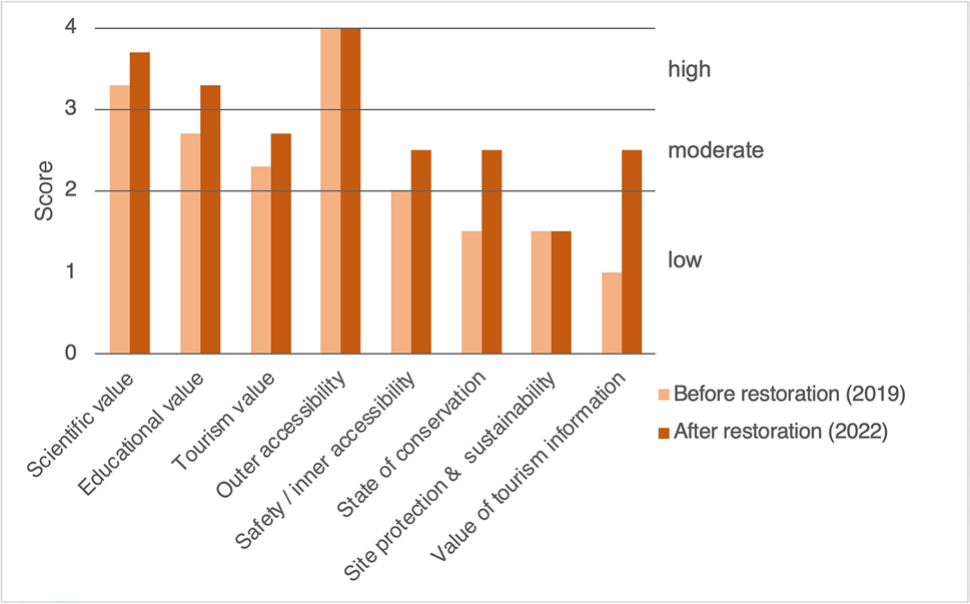
Semiquantitative assessment of the Roses lighthouse geosite before and after the 2020–2021 restoration. Values are in the range 0–4 (0–2: low; 2.1–3: moderate; 3.1–4: high). Adapted from the criteria and method of Suzuki and Takagi (2018)

Multiple methods have been proposed in the last decades for the quantitative or semiquantitative assessment of geosite values and conservation (Brilha 2018). A majority of these methods are based on a list of criteria relevant for the ultimate aims of geoheritage inventory, planning, and/or management (e.g., Bruschi et al. 2011; Fassoulas et al. 2012; Reynard et al. 2015; Brilha 2016; Suzuki and Takagi 2018). Thus, the fundamental criteria used in the different methods do not vary substantially: representativeness and uniqueness of scientific and educational values, tourism potential, and vulnerability and threats regarding geoconservation. It is rather the specific weight given to certain criteria that varies depending on the site typology to be considered or the specific goal (inventory, protection, management) to be achieved. Some methods represent adaptations or small modifications of more generic ones. This is the case, for instance, of the methods adapted and extended to urban geosites by Kubalíková et al. (2019) and Vegas and Díez-Herrero (2021). In the case of Roses Lighthouse periurban geosite, we have applied Suzuki and Takagi (2018) criteria with some modifications, resulting into a semiquantitative assessment of eight parameters (summarized in Fig. 10).

Scientific, educational, and touristic values have experienced a significant increase after recovering some outcrop exposures, improving access and visibility conditions and implementing the dissemination project. There are still a few big boulders laying on top of some outcrops which should be removed.

The value of on-site and website geological information has switched from almost non-existent to conform a notable geotourism resource, which can still be improved if such information is supplied in several languages.

As for the accessibility criteria, we distinguish between outer and inner accessibility. Outer accessibility refers to how to arrive to a given geosite (walking time from public transport stops or parking lot), while inner accessibility is referred to on-site, “touch the rock” chances (following the concepts of Mikhailenko et al. 2021). The first was already very high before restoration given the periurban character of the geosite and closeness to roads. In contrast, inner accessibility, which we have joined together with safety conditions in a combined scoring parameter, has increased from low to moderate levels thanks to the removal of plants and building of a walkway with a staircase. A future action for enhancing accessibility could be extending the walkway to connect portal 1 and portal 2 (Fig. 9).

State of conservation or integrity has much improved from low to moderate. However, the degradation risk remains high, as reflected by the low index of site protection and sustainability. This is due to the high vulnerability of the outcrops to natural processes and human activities, especially to the combination of a likely increase in visitors’ concurrence and the lack of a specific official protection and regulation of the geosite. Thus, geoconservation of these delicate rock exposures will require further actions such a specific protection law entailing geosite use regulation (including restrictions like the need for permission for scientific sampling) and a regular maintenance plan to avoid endangerment and deterioration.

## Concluding Remarks

The Roses Lighthouse geosite is a paradigm of a periurban locality with valuable geological outcrops (recognized by the international scientific community since the early 1980 decade) which suffered transformation into a strongly degraded site due to natural processes and urban pressure (decades of 1990–2010). It has been recently (2021) partially restored with geoconservation criteria, after an effective cooperation between professionals and academics of geology and landscape architecture. The results were an important improvement in outcrop access and visibility and dissemination of their scientific and educational values to the general public throughout on-site and website information. However, we consider that these actions, performed with limited financial resources, are insufficient. The following actions are needed to guarantee the full conservation and sustainability of the site:

- Further recover of outcrop exposures by removal of boulders and also further improvement of inner accessibility and dissemination materials.
- Creation of a specific figure of legal protection which should be based on geconservation and historical-cultural criteria, independent of the degree of anthropization and not subjected to naturality or aesthetic beauty restrictions. Scientific, educational and tourist use of the geosite should also be properly regulated.
- Design and implementation of a strong sustainable conservation management plan in order to ensure a balance between tourist/leisure developments and geoconservation needs, preventing deterioration due to high visitor concurrence.

We believe that the issues emerged from this case study can be extrapolated to other geosites located in periurban areas or to similar contexts where anthropization prevails.
